# Supplementary material for: Role of Glycolysis/Gluconeogenesis and HIF-1 Signaling Pathways in Rats with Dental Fluorosis Integrated Proteomics and Metabolomics Analysis
Source: Int J Mol Sci. 2022 Jul 27;23(15):8266. doi: 10.3390/ijms23158266 (PMC9332816; doi:10.3390/ijms23158266)
Supplement: Supplementary file 1 [file ijms-23-08266-s001.zip › Supplementary figure.pdf]

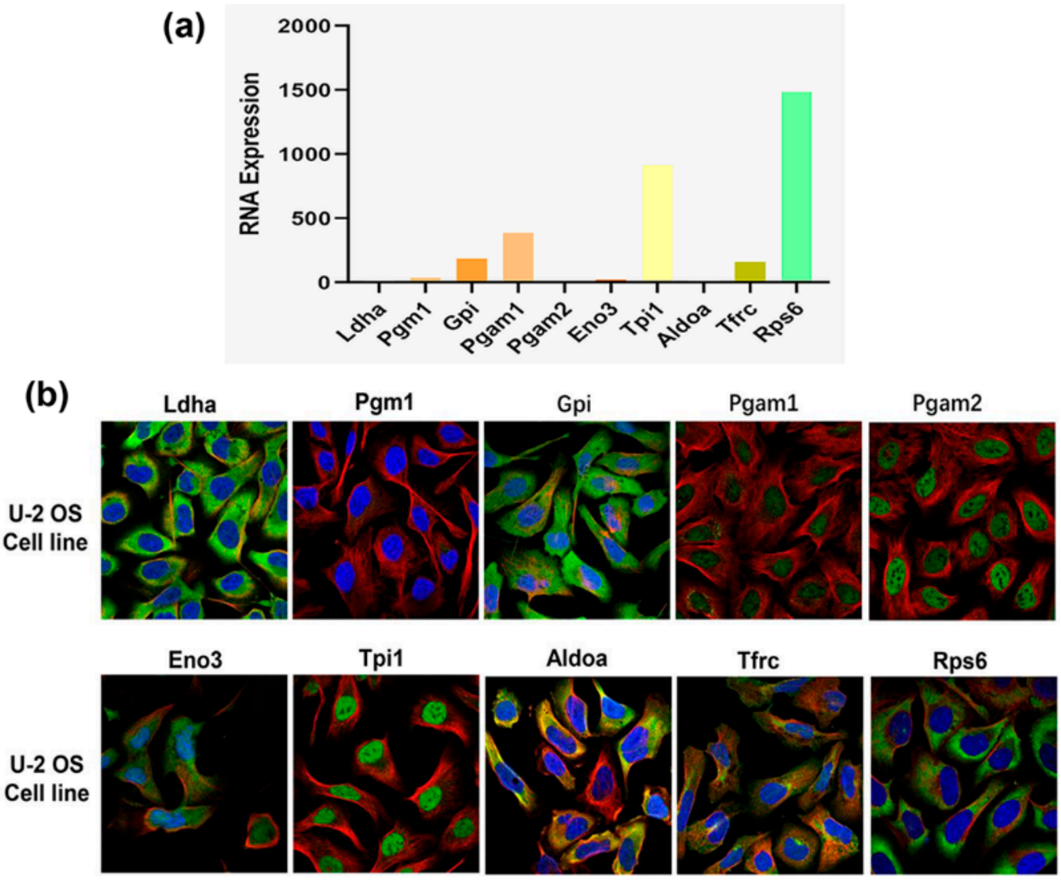

Figure S1. The expression (**a**) and distribution (**b**) of key proteins in the U2-OS cell lines. The distribution and expression of fluorine-related proteins were obtained from the Human Protein Atlas Database (https://www.proteinatlas.org/). In the U-2 OS cell line, green represents the target protein, blue represents the nucleus, and red represents microtubules. The validation images were all from the Human Protein Atlas Database [39].
