# Supplementary material for: Role of Glycolysis/Gluconeogenesis and HIF-1 Signaling Pathways in Rats with Dental Fluorosis Integrated Proteomics and Metabolomics Analysis
Source: Int J Mol Sci. 2022 Jul 27;23(15):8266. doi: 10.3390/ijms23158266 (PMC9332816; doi:10.3390/ijms23158266)
Supplement: Supplementary file 1 [file ijms-23-08266-s001.zip › Supplementary tables.pdf]

Table S1. The expression of differentially expressed proteins between dental fluorosis group (n=6) and control group (n=8)

Accession	Gene Name	Dental fluorosis	Control	*P* value	FC
Q6IFU9	Krt16	267.9±8.2	22.1±8.5	1.01×10^{-15}	12.14
Q6IFV1	Krt14	249.9±6.8	28.7±3.0	6.37×10^{-18}	8.70
Q6IG05	Krt75	240.8±5.3	32.9±6.0	$7.39 \times 10\text{-}^{17}$	7.31
Q63532	Sprr1a	224.7±18.8	50.2±17.4	4.89×10^{-10}	4.47
Q10758	Krt8	170.6±15.9	38.9±3.3	2.54×10^{-11}	4.39
Q6P6Q2	Krt5	208.7±4.3	48.4±2.2	1.79×10^{-18}	4.31
Q6IFW6	Krt10	207.8±6.0	48.6±2.6	7.19×10^{-17}	4.28
A0A0G2JU43	LOC100361920	47.9±7.5	13.3±1.9	2.73×10^{-8}	3.61
P06760	Gusb	198.7±7.7	57.8±5.7	4.62×10^{-14}	3.44
A0A0G2JWX4	Krt2	188.1±3.9	58.4±2.4	1.65×10^{-17}	3.22
Q6IMF3	Krt1	181.0±3.5	59.4±2.5	1.97×10^{-17}	3.05
A0A0G2JXH6	Krt73	176.7±5.1	60.4±2.6	7.63×10^{-16}	2.92
P22509	Fbl	162.0±12.2	64.8±9.0	7.97×10^{-10}	2.50
P0DMW0	Hspa1a	49.1±5.6	21.1±3.2	5.25×10^{-8}	2.33
P06765	Pf4	140.5±5.6	64.9±7.1	5.75×10^{-11}	2.16
Q08163	Cap1	73.2±2.6	34.1±2.1	7.08×10^{-13}	2.15
Q4FZU2	Krt6a	153.8±5.9	72.8±4.8	2.27×10^{-12}	2.11
A0A0H2UHE4	Reg3b	137.3±2.2	65.6±1.7	5.40×10^{-17}	2.09
A0A0G2JV65	Ywhaz	72.9±4.4	34.9±3.3	3.79×10^{-10}	2.09
Q6B345	S100a11	41.5±6.0	21.0±3.7	4.18×10^{-6}	1.98
M0R757	LOC10036043	39.4±12.0	19.9±3.5	8.42×10^{-4}	1.98
G3V852	Tln1	102.5±3.2	52.6±1.9	1.06×10^{-13}	1.95
Q63610	Tpm3	59.6±4.7	31.2±6.0	6.18×10^{-7}	1.91
P34058	Hsp90ab1	62.7±2.1	33.0±2.3	1.18×10^{-11}	1.90
P09495	Tpm4	55.6±7.4	29.4±3.9	1.74×10^{-6}	1.89
P07150	Anxa1	32.7±5.8	17.4±3.9	7.19×10^{-5}	1.88
P62755	Rps6	35.5±12.0	19.5±8.4	1.18×10^{-2}	1.82
P62260	Ywhae	67.9±2.5	38.5±3.3	4.22×10^{-10}	1.76
P63018	Hspa8	71.8±1.8	41.1±2.1	2.01×10^{-12}	1.75

P04642	Ldha	24.9±10.5	14.3±4.0	2.14×10^{-2}	1.74
P05197	Eef2	59.5±8.6	34.3±19.7	1.30×10^{-2}	1.74
Q62636	Rap1b	77.2±1.8	46.2±1.8	5.54×10^{-13}	1.67
Q66HT1	Aldob	135.5±3.1	81.2±5.0	2.63×10^{-11}	1.67
D3ZJP6	Myo10	54.7±4.2	34.3±3.0	1.99×10^{-7}	1.60
B2RZ72	Arpc4	60.0±9.9	38.0±5.4	1.74×10^{-4}	1.58
P42930	Hspb1	43.6±17.1	27.8±5.3	2.82×10^{-2}	1.57
A0A0G2JSH5	Alb	122.3±1.9	79.1±1.9	2.05×10^{-14}	1.55
B0BMS8	Myl9	34.2±4.2	22.2±2.7	3.14×10^{-5}	1.54
Q5XFX0	Tagln2	47.7±4.0	31.2±2.3	4.19×10^{-7}	1.53
P42854	Reg3g	123.5±3.1	80.9±3.4	1.76×10^{-11}	1.53
G3V6P7	Myh9	53.3±5.4	35.1±4.5	1.86×10^{-5}	1.52
G3V7J7	Eif5a2	59.9±5.2	39.6±4.4	4.14×10^{-6}	1.51
D4A367	Rbck1	35.0±11.5	23.3±6.5	3.22×10^{-2}	1.50
P38652	Pgm1	134.5±4.7	89.7±3.5	1.12×10^{-10}	1.50
D3ZYE2	RGD1565617	119.2±3.7	79.5±2.5	1.47×10^{-11}	1.50
Q6P6V0	Gpi	64.7±3.6	43.3±3.2	5.78×10^{-8}	1.50
P25113	Pgam1	66.3±3.2	44.9±4.7	6.13×10^{-7}	1.48
B2GV99	Myl6	30.8±7.1	20.9±3.0	3.92×10^{-3}	1.47
A0A0G2JSP8	Ckm	137.8±4.6	95.0±3.5	1.50×10^{-10}	1.45
P16290	Pgam2	137.8±5.9	96.3±4.0	2.20×10^{-9}	1.43
P62963	Pfn1	86.2±6.1	60.6±6.9	1.14×10^{-5}	1.42
Q6NYB7	Rab1A	36.0±8.5	25.5±5.4	1.50×10^{-2}	1.41
A0A0G2K5U9	Actn4	39.1±4.2	27.8±5.5	1.26×10^{-3}	1.40
P15429	Eno3	129.2±8.5	92.0±4.0	1.35×10^{-7}	1.40
A0A0G2K3K2	Actb	62.0±5.3	44.3±4.4	1.85×10^{-5}	1.40
P62986	Uba52	59.0±4.3	42.6±4.4	1.51×10^{-5}	1.39
P61589	Rhoa	40.4±6.4	29.2±8.1	1.70×10^{-2}	1.38
A0A096MK30	Msn	46.7±7.4	34.0±2.3	6.06×10^{-4}	1.37
A0A0G2K8V2	Vcl	86.8±2.2	63.2±2.0	9.39×10^{-11}	1.37
A0A0G2K5U5	Pigr	106.3±4.7	77.5±4.2	4.63×10^{-8}	1.37

Q9EQS0	Taldo1	70.3±5.8	51.7±4.3	1.69×10^{-5}	1.36
P68370	Tuba1a	39.2±6.8	28.9±2.3	1.60×10^{-3}	1.36
D3ZJW6	rCG_21066	124.9±2.5	93.5±1.9	4.96×10^{-12}	1.34
P69897	Tubb5	35.3±8.1	26.5±3.7	1.86×10^{-2}	1.33
F1LR92	Serpina3m	112.8±1.0	85.5±1.5	8.68×10^{-14}	1.32
P09811	Pygl	117.2±9.6	89.0±8.0	6.47×10^{-5}	1.32
P45592	Cfl1	54.4±6.0	41.3±10.7	2.00×10^{-2}	1.32
F1M7M1	Lalba	114.1±2.4	86.9±3.9	4.06×10^{-9}	1.31
C0JPT7	Flna	40.2±5.2	31.0±2.7	9.32×10^{-4}	1.30
P55797	Apoc4	117.0±1.5	90.3±1.6	7.45×10^{-13}	1.30
P07632	Sod1	92.9±5.0	71.8±3.7	9.25×10^{-7}	1.30
P10111	Ppia	42.4±4.8	32.8±4.0	1.64×10^{-3}	1.29
P63269	Actg2	59.1±5.8	45.9±3.7	2.23×10^{-4}	1.29
P48500	Tpi1	63.6±5.8	49.5±4.7	2.95×10^{-4}	1.29
B2RZ27	Sh3bgrl3	47.7±6.5	37.3±5.6	7.55×10^{-3}	1.28
P31211	Serpina6	121.4±1.5	95.3±2.1	6.28×10^{-12}	1.27
P16617	Pgk1	58.6±6.1	46.0±3.4	3.39×10^{-4}	1.27
Q9QZ76	Mb	115.8±2.2	91.3±3.6	5.67×10^{-9}	1.27
Q5M8C3	Serpina4	115.7±2.1	92.2±2.4	2.21×10^{-10}	1.26
Q99376	Tfrc	109.9±1.9	88.1±2.7	1.03×10^{-9}	1.25
Q6TXF3	Dbi	114.7±3.7	92.3±4.0	1.87×10^{-7}	1.24
Q66HD0	Hsp90b1	52.9±7.6	42.6±2.8	3.90×10^{-3}	1.24
G3V826	Tkt	83.9±4.9	67.7±7.7	6.94×10^{-4}	1.24
Q5XI38	Lcp1	101.7±2.2	82.2±2.2	1.51×10^{-9}	1.24
Q99J82	Ilk	31.4±3.8	25.5±5.1	3.49×10^{-2}	1.23
P04785	P4hb	59.5±2.4	48.6±2.1	8.80×10^{-7}	1.22
A0A0G2JSK1	Serpina3c	112.7±1.2	92.2±1.1	4.11×10^{-13}	1.22
P04041	Gpx1	117.3±3.3	96.2±2.8	1.99×10^{-8}	1.22
Q6T487	Actn1	36.1±6.6	29.8±3.3	3.82×10^{-2}	1.21
A0A0G2K975	RGD1564614	91.7±2.9	111.2±3.7	1.72×10^{-7}	0.82
P05545	Serpina3k	96.3±1.1	117.6±0.8	1.97×10^{-14}	0.82

D3ZAF5	Postn	38.2±7.6	46.7±3.9	1.81×10^{-2}	0.82
P01026	C3	84.5±4.0	107.1±5.0	1.04×10^{-6}	0.79
P28073	Psmb6	83.0±2.2	105.6±6.2	2.05×10^{-6}	0.79
D3ZJF8	Fcgbp	81.9±2.7	106.3±3.0	2.40×10^{-9}	0.77
D3ZLE6	RT1-CE7	90.5±4.6	117.7±6.7	1.98×10^{-6}	0.77
Q6P734	Serping1	90.1±0.5	118.0±2.1	7.59×10^{-13}	0.76
P06866	Hp	88.5±1.5	125.8±6.5	1.13×10^{-8}	0.70
P59996	Pcsk9	82.2±2.9	117.2±4.2	6.12×10^{-10}	0.70
Q4V8L0	Timp3	16.4±5.7	23.9±3.0	7.65×10^{-3}	0.69
P05065	Aldoa	67.7±2.2	98.7±2.8	3.32×10^{-11}	0.69
P08649	C4	83.9±1.2	124.6±0.6	6.68×10^{-18}	0.67
A0A0G2K542	Ugp2	77.0±2.7	117.4±2.9	4.13×10^{-12}	0.66
Q6MG90	C4b	77.8±1.0	125.9±2.6	1.45×10^{-14}	0.62
P15978	RT1-Aw2	74.5±6.0	120.9±6.8	1.62×10^{-8}	0.62
P02764	Orm1	81.6±1.4	133.7±0.8	2.45×10^{-18}	0.61
P01048	Map1	77.5±2.7	141.7±3.5	9.88×10^{-14}	0.55
Q5M8C6	Fgl1	75.7±1.8	142.6±3.7	2.82×10^{-14}	0.53
F7FAY5	LOC360919	67.1±1.6	129.1±2.5	1.40×10^{-15}	0.52
A0A0G2KA54	Kng1	74.3±2.5	144.7±2.5	1.88×10^{-15}	0.51
G3V885	Myh6	15.6±7.5	34.2±3.5	4.27×10^{-5}	0.46
P11980	Pkm	45.1±2.6	101.2±4.8	7.05×10^{-12}	0.45
A0A0G2KA12	Kif1b	53.0±13.8	149.2±16.6	7.71×10^{-8}	0.36
G3V8V3	Pygm	41.2±2.0	183.4±5.8	5.34×10^{-16}	0.22
P06238	A2m	23.5±5.6	212.7±7.8	2.55×10^{-15}	0.11

Note: The expression of differentially expressed proteins were presented mean ± standard deviation, the fold change (FC) >1.2 or < 0.83 and $P < 0.05$ were considered as significant.

Table S2. The expression of differentially expressed metabolites between dental fluorosis mice (n=6) and normal mice (n=8)

Metabolite name	HMDB	Dental fluorosis	Control	FC	P-value
Pinitol	HMDB0034219	9.57±8.57	21.54±6.34	0.44451	0.01081
L-glutamine	HMDB0000641	$(5.17\pm3.57)\times10^{-2}$	$(1.57\pm1.65)\times10^{-2}$	3.30151	0.02593
Indole-3-propionic acid	HMDB0002302	169.72±83.97	75.56±22.92	2.2463	0.00988
(s)-3-hydroxyisobutyric acid	HMDB0000023	$(4.22\pm1.31)\times10^{-3}$	$(6.65\pm1.22)\times10^{-3}$	0.6353	0.00389
Isomaltose	HMDB0002923	70.09±42.22	32.40±18.27	2.16341	0.04171
Ethanolamine	HMDB0000149	$(1.30\pm0.38)\times10^{-2}$	$(8.41\pm1.85)\times10^{-3}$	1.54347	0.01101
Indolelactic acid	HMDB0000671	12.96±7.29	6.50±1.38	1.99274	0.02911
Saccharic acid	HMDB0000663	4.27±1.30	2.69±1.23	1.58666	0.03862
4-methylvaleric acid	HMDB0000689	$(1.90\pm0.94)\times10^{-3}$	$(1.09\pm0.12)\times10^{-3}$	1.73691	0.03241
Hydrocinnamic acid	HMDB0000764	1.75±0.39	1.29±0.14	1.35703	0.00949
Hypoxanthine	HMDB0000157	$(2.63\pm0.86)\times10^{-3}$	$(1.91\pm0.24)\times10^{-3}$	1.3779	0.04156
L-methionine	HMDB0000696	$(7.49\pm2.04)\times10^{-3}$	$(5.70\pm0.57)\times10^{-3}$	1.31455	0.03424
N-methylalanine	HMDB0094692	$(1.35\pm0.27)\times10^{-2}$	$(1.07\pm0.12)\times10^{-2}$	1.25978	0.02278
Octanoate radical	HMDB0062511	$(8.54\pm1.13)\times10^{-3}$	$(7.44\pm0.20)\times10^{-3}$	1.1479	0.01789

Note: The expression of differentially expressed metabolites were presented mean±standard deviation, the fold change (FC) >1.2 or < 0.83 and P < 0.05 were considered as significant.
